# High Frequency of Self-Diagnosis and Self-Treatment in a Nationally Representative Survey about Superficial Fungal Infections in Adults—United States, 2022

**DOI:** 10.3390/jof9010019

**Published:** 2022-12-22

**Authors:** Kaitlin Benedict, Jeremy A. W. Gold, Karen Wu, Shari R. Lipner

**Affiliations:** 1 Mycotic Diseases Branch, Division of Foodborne, Waterborne, and Environmental Diseases, National Center for Emerging and Zoonotic Infectious Diseases, Centers for Disease Control and Prevention, Atlanta, GA 30329, USA; 2 Department of Dermatology, Weill Cornell Medicine, New York, NY 10021, USA

**Keywords:** mycoses, tinea, tinea unguium, tinea corporis, ringworm, onychomycosis, public education, antifungals, United States

## Abstract

Data about the prevalence, diagnosis, treatment, and public knowledge of superficial fungal infections in the United States are scarce. These infections are a growing concern given the emergence of antifungal drug resistance. We analyzed data from a national survey of nearly 6000 U.S. adults. Overall, 114 (2.7%) participants reported having ringworm and 415 (10.0%) reported a fungal nail infection in the past 12 months; 61.4% of participants with any superficial fungal infection were self-diagnosed. Most patients (55.5%) used over-the-counter antifungals. The common nature of superficial fungal infections and the high rates of self-diagnosis and treatment indicate that community education about these infections should be considered a public health priority.

## 1. Introduction

Superficial fungal infections are commonly seen in dermatologic practice, are underrecognized public health problems, and are concerning given the emergence of antifungal-drug-resistant tinea and onychomycosis [1,2,3]. However, dermatologists frequently see patients who suspect that they have ringworm or fungal nail infections who are subsequently diagnosed with eczema or traumatic onycholysis, respectively [4]. We analyzed data from an online survey of U.S. adults to estimate the prevalence, diagnosis, treatments, and knowledge of terminology of superficial fungal infections.

## 2. Methods

We analyzed data from Porter Novelli’s summer 2022 ConsumerStyles survey. The self-administered online survey covered various health topics, including questions about ringworm prevalence, treatment, and complications (Supplemental Table S1). Survey participants were randomly recruited from Ipsos’ nationally representative KnowledgePanel^®^ by mail using address-based probability sampling. Participants were provided with a laptop or tablet computer and Internet access if needed. The survey weights were designed to match the March 2021 U.S. Current Population Survey proportions in terms of gender by age, household income, race/ethnicity, household size, education, census region, metropolitan status, and parental status of children 11–17 years old.

We used weighted descriptive and bivariate analyses to examine demographics, co-morbidities, treatments, and complications associated with having a superficial fungal infection in the past year.

## 3. Results

The survey was sent to 5,990 adults, with a 69.4% response rate (N = 4,156). Overall, 114 (2.7%) participants reported having ringworm, 415 (10.0%) reported a fungal nail infection, and 37 reported both in the past 12 months (Table 1). Participants with ringworm vs. without were more frequently men (64.0% vs. 48.2%, *p* = 0.008), and those with fungal nail infection vs. without were older (mean 54.7 vs. 47.3 years, *p* < 0.001) and more likely to have diabetes (18.1% vs. 10.8%, *p* < 0.001). Fungal nail infection prevalence varied by race/ethnicity (*p* < 0.001) and was highest among Hispanic participants (14.2%).

Among 492 participants with any superficial fungal infection, 38.6% were healthcare provider (HCP)-diagnosed. Most patients used over-the-counter antifungals (55.5%); 18.3% used alternative or natural treatments (Table 2). Among 408 participants who used any treatment, 24.0% reported that treatment was ineffective, and 3.4% experienced side effects. In total, 28.7% reported complications. Overall awareness of superficial fungal infection terminology (e.g., “ringworm,” “athlete’s foot”) was 86.4%, with greater awareness among older adults, non-Hispanic whites, and persons with higher educational levels and underlying health conditions (Supplemental Table S2).

## 4. Discussion

Our study shows that superficial fungal infections are relatively common, with high rates of self-diagnosis and self-treatment. Fungal nail infection prevalence in this study was consistent with others (2–14%) [5,6]. Although overall knowledge of superficial fungal infection nomenclature was encouraging, the high treatment failure rate highlights a lack of awareness about the importance of HCP diagnosis and management of superficial fungal infections and the need for comprehensible public education material [7]. The modest use of natural or alternative treatments also supports the need for public education on evidenced-based treatments [8].

Despite the high rates of self-diagnosis, factors associated with superficial fungal infections (e.g., older age, diabetes, and Hispanic ethnicity for fungal nail infection and male sex for ringworm) were generally consistent with other studies [6,9,10]. Other previously reported risk factors for onychomycosis include nail trauma, immunosuppression, and tinea pedis [11]. Risk factors for ringworm can vary depending on the body site affected, which we were unable to evaluate due to small sample sizes. We were also not able to evaluate possible exposure sources or potentially modifiable risk factors, such as contact with contaminated surfaces or infected persons or animals [12].

Potential recall bias is this study’s main limitation. Prevalence estimates could be affected by participants’ disease misclassification or if HCPs diagnosed patients without confirmatory testing, which unfortunately is a common occurrence in clinical practice [6]. The high reported treatment failure rate that we observed is likely confounded by severity and care-seeking, and could reflect an incorrect treatment type, poor treatment adherence, antifungal resistance, or incorrect diagnosis, which are plausible given the high self-diagnosis rate. Surprisingly, rates of treatment failure were higher among patients with HCP-diagnosed cases vs. those with self-diagnosed cases. We suspect that this finding might be because patients with more severe infections are more likely to be seen by a physician rather than self-treating at home. The finding that side effects were more common in the HCP-treated group vs. self-treated group might be because the HCP group were prescribed oral antifungals in some cases. Lastly, we were unable to investigate fungal species with this survey, which is a key factor informing testing and treatment strategies, as antifungal resistance is a concern in both dermatophyte and non-dermatophyte infections [13].

In sum, our study highlights the extensive self-diagnosis and treatment of U.S. superficial fungal infections. Diagnosis by a healthcare provider, combined with confirmatory laboratory testing for superficial fungal infections, is important for appropriate treatment selection [14,15]. Given their high prevalence and the potential to contribute to antifungal resistance through indiscriminate use of over-the counter antifungals, community education on the proper diagnosis and treatment of superficial fungal infections should be considered as a public health priority.

## Figures and Tables

**Table 1:** Demographic characteristics and health-related features associated with having ringworm or fungal nail infection in the past 12 months—Porter Novelli Summer ConsumerStyles Survey, United States, 2022.

	Ringworm ^1,2^			Fungal Nail Infection ^1^		
	Yes (n = 114)	No (n = 4027)		Yes (n = 415)	No (n = 3727)	
Characteristic	n (%)	n (%)	*p*-Value	n (%)	n (%)	*p*-Value
Mean, median age in years (IQR)	44.5, 43.6 (27.6–57.4)	48.1, 47.3 (31.9–62.4)	0.066	54.7, 57.1 (42.0–68.3)	47.3, 45.7 (31.5–61.5)	<0.001
Age category in years			0.265			<0.001
18–34	42 (36.5%)	1168 (29.0%)		82 (19.7%)	1128 (30.3%)	
35–44	17 (14.8%)	671 (16.7%)		41 (9.8%)	647 (17.4%)	
45–54	23 (20.1%)	626 (15.6%)		69 (16.6%)	580 (15.6%)	
55–64	16 (13.8%)	669 (16.6%)		82 (19.8%)	602 (16.2%)	
65 and older	17 (15.2%)	893 (22.2%)		142 (34.1%)	769 (20.6%)	
Gender ^3^			0.008			0.093
Male	73 (64.0%)	1934 (48.2%)		220 (53.0%)	1788 (48.0%)	
Female	41 (36.0%)	2082 (51.8%)		194 (46.7%)	1929 (51.8%)	
Race/ethnicity			0.281			<0.001
White, non-Hispanic	70 (61.4%)	2524 (62.7%)		264 (63.6%)	2330 (62.5%)	
Black, non-Hispanic	9 (7.6%)	485 (12.0%)		33 (7.9%)	460 (12.3%)	
Other/multiple race, non-Hispanic	8 (7.0%)	350 (8.7%)		17 (4.7%)	338 (9.1%)	
Hispanic	28 (24.5%)	668 (16.6%)		99 (23.8%)	598 (16.0%)	
Education			0.053			0.386
High school or less	31 (27.2%)	1536 (38.1%)		147 (35.4%)	1420 (38.1%)	
Some college or more	84 (73.7%)	2,491 (61.9%)		267 (64.3%)	2307 (61.9%)	
Mean, median number of people in household (IQR)	3.3, 2.3 (1.4–3.8)	2.9, 2.0 (1.3–3.4)	0.128	2.8, 1.8 (1.3–2.9)	2.9, 2.1 (1.3–3.4)	0.163
Have children in household	36 (31.6%)	1198 (29.7%)	0.740	95 (22.9%)	1139 (30.6%)	0.010
Employed	86 (75.4%)	2394 (59.4%)	0.002	220 (53.0%)	2260 (60.6%)	0.008
Household income			0.697			0.331
$0 to $24,999	16 (14.0%)	515 (12.8%)		63 (15.2%)	468 (12.6%)	
$25,000 to $74,999	42 (36.8%)	1338 (33.2%)		143 (34.5%)	1237 (33.2%)	
$75,000 to $149,999	37 (32.5%)	1253 (31.1%)		130 (31.3%)	1159 (31.1%)	
$150,000 or more	20 (17.5%)	922 (22.9%)		79 (19.0%)	861 (23.1%)	
Mean, median number of healthcare provider visits in the past 12 months (IQR)	5.5, 2.3 (0.4–4.5)	4.4, 1.8 (0.3–4.4)	0.305	5.1, 2.8 (0.8–5.7)	4.3, 1.7 (0.2–4.2)	0.088
Health conditions in the past 12 months						
Diabetes	17 (14.9%)	460 (11.4%)	0.273	75 (18.1%)	402 (10.8%)	<0.001
No health conditions	22 (19.3%)	981 (24.4%)	0.322	51 (12.3%)	952 (25.5%)	<0.001
Community type			0.809			0.010
Urban	39 (34.2%)	1421 (35.3%)		124 (29.9%)	1336 (35.8%)	
Rural	23 (20.2%)	693 (17.2%)		79 (19.0%)	637 (17.1%)	
Suburban	52 (45.6%)	1910 (47.4%)		212 (51.1%)	1751 (47.0%)	
Census region			0.676			0.621
Northeast	24 (21.4%)	690 (17.1%)		75 (18.1%)	640 (17.2%)	
Midwest	26 (23.2%)	831 (20.6%)		75 (18.1%)	781 (21.0%)	
South	38 (33.4%)	1543 (38.3%)		158 (38.1%)	1423 (38.2%)	
West	26 (22.4%)	963 (23.9%)		107 (25.8%)	882 (23.7%)	

IQR = interquartile range. ^1^ n missing response = 18. ^2^ Body sites affected were: foot in 42 (36.8%), groin/inner thighs/buttocks in 29 (25.4%), hand in 13 (11.4%), face in 9 (7.9%), scalp in 5 (4.4%), somewhere else on the body in 33 (28.9%), and don’t know/don’t remember in 8 (7.0%). ^3^ Eleven respondents without ringworm, 1 respondent with fungal nail infection, and 10 respondents without fungal nail infection answered “prefer to self-describe”.

**Table 2:** Treatments for and complications of superficial fungal infections (ringworm and fungal nail infections)—Porter Novelli Summer ConsumerStyles Survey, United States, 2022 ^1^.

	Total (n = 492)	Diagnosed by a Healthcare Provider ^2^ (n = 190)	Not Diagnosed by a Healthcare Provider (n = 302)	
Treatments	n (%)	n (%)	n (%)	*p*-Value
Non-prescription cream, powder, etc.	273 (55.5%)	69 (36.3%)	203 (67.2%)	<0.001
Prescription cream, powder, etc.	110 (22.4%)	88 (46.3%)	23 (7.6%)	<0.001
Prescription medicine taken by mouth	59 (12.0%)	50 (26.3%)	9 (3.0%)	<0.001
Alternative or natural treatment	90 (18.3%)	25 (13.2%)	65 (21.5%)	0.030
None of the above	84 (17.1%)	24 (12.6%)	60 (19.9%)	0.085
Side effects of treatment (n = 408)	14 (3.4%)	11 (6.7%)	3 (1.2%)	0.003
Treatment did not cure infection (n = 408)	98 (24.0%)	52 (31.5%)	46 (19.0%)	0.008
**Complications**	141 (28.7%)	54 (28.4%)	88 (29.1%)	0.900
Bacterial infection (“cellulitis”)	37 (7.5%)	20 (10.5%)	17 (5.6%)	0.138
Permanent skin or nail damage	119 (24.2%)	39 (20.5%)	80 (26.5%)	0.212

^1^ We did not observe notable differences in demographic or health-related factors among people with healthcare provider-diagnosed vs. self-diagnosed infections. ^2^ Three respondents who reported having ringworm diagnosed by a healthcare provider also reported self-diagnosed ringworm, and 9 respondents who reported having a fungal nail infection diagnosed by a healthcare provider also reported a self-diagnosed fungal nail infection.

## Data Availability

The data used in this study are available from Porter Novelli, https://www.porternovelli.com/.

## Supplementary Materials

The following supporting information can be downloaded at: https://www.mdpi.com/article/10.3390/jof9010019/s1, Table S1: Ringworm and fungal nail infection questions and response options—Porter Novelli Summer ConsumerStyles Survey, United States, 2022; Table S2: Ever heard of any of the fungal infection terms listed on the survey—Porter Novelli Summer ConsumerStyles Survey, United States, 2022.
