# Supplementary material for: High Frequency of Self-Diagnosis and Self-Treatment in a Nationally Representative Survey about Superficial Fungal Infections in Adults—United States, 2022
Source: J Fungi (Basel). 2022 Dec 22;9(1):19. doi: 10.3390/jof9010019 (PMC9860956; doi:10.3390/jof9010019)
Supplement: Supplementary file 1 [file jof-09-00019-s001.zip › jof-2040963-supplementary.pdf]

Supplemental Table S1: Ringworm and fungal nail infection questions and response options—Porter Novelli Summer ConsumerStyles Survey, United States, 2022

**Question**	**Response options**
1. Have you ever heard of the following infections?<br>*Select all that apply.*	• Ringworm<br>• Tinea<br>• Athlete's foot<br>• Jock itch<br>• Dermatophytosis<br>• Fungal nail infection<br>• None of these
2. In the past 12 months, have you had any of the following infections? Ringworm is sometimes known as "tinea," "athlete's foot," or "jock itch." *Select all that apply.*	• Ringworm, not diagnosed by a healthcare provider<br>• Ringworm, diagnosed by a healthcare provider<br>• Fungal nail infection, not diagnosed by a healthcare provider<br>• Fungal nail infection, diagnosed by a healthcare provider<br>• None of these<br>• Don't know/don't remember
3. *Among respondents who had ringworm:*<br>In the past 12 months, which of the following places on your body did you have ringworm?<br>*Select all that apply.*	• Foot ("athlete's foot")<br>• Groin/inner thighs/buttocks ("jock itch")<br>• Scalp<br>• Face<br>• Hand<br>• Somewhere else on the body<br>• Don't know/don't remember
4. *Among respondents who had ringworm:*<br>Which of the following antifungal treatments did you use to treat your ringworm infection?<br>*Select all that apply.*	• Non-prescription cream, powder, etc.<br>• Prescription cream, powder, etc.<br>• Prescription medicine taken by mouth<br>• Alternative or natural treatment<br>• None of the above
5. *Among respondents who had a fungal nail infection:*<br>Which of the following antifungal treatments did you use to treat your fungal nail infection?<br>*Select all that apply.*	• Non-prescription cream, powder, etc.<br>• Prescription cream, powder, etc.<br>• Prescription medicine taken by mouth<br>• Alternative or natural treatment<br>• None of the above
6. *Among respondents who had ringworm or a fungal nail infection*:<br>Which of the following issues or complications did you have as a result of your infection?<br>*Select all that apply.*	• Bacterial infection ("cellulitis")<br>• Permanent skin or nail damage<br>• Side effects from treatment<br>• Treatment did not cure infection<br>• None of the these

Supplemental Table S2: Ever heard of any of the fungal infection terms listed on the survey[1]—Porter Novelli Summer ConsumerStyles Survey, United States, 2022

	**Yes (n = 3,573)**	**No (n = 567)**	
**Characteristic**	**n (%)**	**n (%)**	***p*-value**
Mean, median age in years (IQR)	49.4, 49.2 (33.5–63.4)	39.6, 40.0 (24.9–49.4)	<0.001
Age category in years			<0.001
18–34	936 (26.2%)	267 (47.1%)	
35–44	570 (16.0%)	116 (20.5%)	

45–54	587 (16.4%)	62 (10.9%)	
55–64	627 (17.5%)	59 (10.4%)	
65 and older	851 (23.8%)	63 (11.1%)	
Gender[2]			0.646
Male	1,721 (48.3%)	279 (49.6%)	
Female	1,844 (51.7%)	283 (50.3%)	
Race/ethnicity			0.005
White, non-Hispanic	2,293 (64.2%)	304 (53.6%)	
Black, non-Hispanic	419 (11.7%)	74 (13.1%)	
Other/multiple race, non-Hispanic	289 (8.1%)	65 (11.5%)	
Hispanic	571 (16.0%)	124 (21.9%)	
Education			0.013
High school or less	1,314 (36.8%)	251 (44.2%)	
Some college or more	2,259 (63.2%)	316 (55.7%)	
Mean, median number of people in household (IQR)	2.9, 2.0 (1.8–3.3)	3.2, 2.5 (1.4–3.8)	0.003
Have children living in household	1,026 (28.7%)	207 (36.5%)	0.006
Employed	2,117 (59.2%)	357 (63.0%)	0.215
Household income			0.412
$0 to $24,999	441 (12.3%)	89 (15.7%)	
$25,000 to $74,999	1,200 (33.6%)	179 (31.6%)	
$75,000 to $149,999	1,116 (31.2%)	174 (30.7%)	
$150,000 or more	815 (22.8%)	125 (22.0%)	
Mean, median number of healthcare provider visits in the past 12 months (IQR)	4.4, 1.9 (0.4–4.5)	4.6, 1.2 (0.0–3.5)	0.769
Health conditions in the past 12 months			
Diabetes	440 (12.3%)	36 (6.3%)	<0.001
No health conditions	767 (21.5%)	229 (40.4%)	<0.001
Metropolitan Statistical Area category			0.882
Non-metropolitan	480 (13.4%)	74 (13.1%)	
Metropolitan	3,093 (86.6%)	493 (86.9%)	
Community type			0.003
Urban	1,225 (34.3%)	230 (40.6%)	
Rural	656 (18.4%)	63 (11.1%)	
Suburban	1,689 (47.3%)	273 (48.1%)	
Census region			0.160
Northeast	633 (17.7%)	80 (14.1%)	
Midwest	727 (20.3%)	131 (23.1%)	
South	1,379 (38.6%)	204 (36.0%)	
West	834 (23.3%)	153 (27.0%)	

IQR = interquartile range

[1] n missing response = 13. In total, 3,225 (77.6%) had heard of ringworm, 665 (16.0%) had heard of tinea, 3,432 (82.6%) had heard of athlete's foot, 2,837 (68.3%) had heard of jock itch, 416 (10.0%) had heard of dermatophytosis, 3,044 (73.2%) had heard of fungal nail infection, and 567 (13.6%) had not heard of any of these terms.

[2] Eleven respondents answered "prefer to self-describe".
